# Scalable Transcriptome Preparation for Massive Parallel Sequencing

**DOI:** 10.1371/journal.pone.0021910

**Published:** 2011-07-07

**Authors:** Henrik Stranneheim, Beata Werne, Ellen Sherwood, Joakim Lundeberg

**Affiliations:** 1 Science for Life Laboratory, Division of Gene Technology, School of Biotechnology, Royal Institute of Technology (KTH), Solna, Sweden; 2 Science for Life Laboratory, Department of Biochemistry and Biophysics, Stockholm University, Solna, Sweden; Dana-Farber Cancer Institute, United States of America

## Abstract

**Background:**

The tremendous output of massive parallel sequencing technologies requires automated robust and scalable sample preparation methods to fully exploit the new sequence capacity.

**Methodology:**

In this study, a method for automated library preparation of RNA prior to massively parallel sequencing is presented. The automated protocol uses precipitation onto carboxylic acid paramagnetic beads for purification and size selection of both RNA and DNA. The automated sample preparation was compared to the standard manual sample preparation.

**Conclusion/Significance:**

The automated procedure was used to generate libraries for gene expression profiling on the Illumina HiSeq 2000 platform with the capacity of 12 samples per preparation with a significantly improved throughput compared to the standard manual preparation. The data analysis shows consistent gene expression profiles in terms of sensitivity and quantification of gene expression between the two library preparation methods.

## Introduction

The massively parallel sequencing technologies continue to evolve at a rapid pace increasing the data output and lowering the cost per sample of sequencing [1], [2], [3]. The Illumina HiSeq 2000 and Life Technologies SOLiD4 are massively parallel sequencing technologies capable of generating over 100 Gbp of sequence data per run. This means that the bottleneck is no longer located in the sequence reaction but in the sample preparation and data analysis. As the number of samples that can be included in a sequencing run increases, so does the complexity of the library preparation. To fully exploit the potential of massive parallel sequencing and further reduce the cost per sample it is essential to prepare many samples robustly [4], with high throughput while minimizing the cross contamination risk. Automation of sample preparation can increase the reproducibility, scalability and ease of handling while minimizing the cost, risk of human error and cross contamination between samples [5], [6], [7], [8]. Recently, there have been several publications relating to automation of library preparations [6], [8], [9] using DNA as the input material. With the continuously decreasing cost of sequencing it is becoming more feasible to consider replacing the gene expression microarrays with RNA-Seq as a means to analyse the transcriptome. Compared to microarrays, RNA-Seq data has proven to be less biased, without cross-hybridization and have a greater dynamic range [10], [11], [12], [13]. The increase in sensitivity of RNA-Seq data makes variant detection more powerful. However, to efficiently use the sequencing power when performing transcriptome analysis a robust and scalable automated library preparation using RNA as input material is needed.

In this study, an automated protocol for transcriptome preparation prior to massively parallel sequencing on the Illumina HiSeq 2000 is described. The protocol was used to prepare libraries for single read sequencing enabling digital profiles of gene expression. The protocol utilises ethanol and tetraethylene glycol to precipitate RNA onto carboxylic acid coated paramagnetic beads instead of the standard ethanol precipitation and all standard spin column steps were replaced with precipitation of DNA using polyethylene glycol and sodium chloride as previously described [6]. The automated protocol was evaluated by comparing it to standard manual procedures with respect to sample throughput, robustness, sensitivity and quantification of gene expression.

## Materials and Methods

### Automation of Transcriptome Sample Preparation

The automation of the Illumina mRNA sequencing sample preparation protocol (Cat# RS-930-1001) was set up using a Magnatrix™ 1200 Biomagnetic Workstation (Nordiag ASA, Oslo, Norway). The robust system provides a flexible software, suitable for customized protocols, and the robot is equipped with a 12-tip head with an adjustable magnet capable of running custom made magnetic bead based applications. The robot is also equipped with one Peltier type (4–95°C), regulated heating/cooling station where all enzymatic reactions were performed, and one PCR cooling block (Eppendorf AG, Hamburg, Germany) for storage of heat sensitive reagents. The mRNA sequencing sample preparation begins with a purification of the poly-A containing mRNA molecules by using Sera-mag magnetic oligo(dT) beads, followed by a fragmentation of the purified mRNA molecules using divalent cations under elevated temperature. The fragmentation was followed by a purification of fragmented RNA using ethanol and tetraethylene glycol (EtOH/TEG; Sigma-Aldrich, St. Louis, MO USA) as a precipitation buffer with Dynabeads® MyOne™ carboxylic acid paramagnetic beads (CA-beads; Invitrogen, Carlsbad, CA USA) as solid support (described in paragraph ‘Evaluation of RNA Precipitation using EtOH/TEG and CA-beads’). The purified fragmented RNA was synthesized into cDNA and isolated using precipitation on CA-beads with PEG 6000 (Merck, Whitehouse station, NJ, USA) and NaCl (Merck) as precipitation buffer and eluted in EB buffer (Qiagen, Hilden, Germany) as previously described [6]. The overhang of the cDNA samples were polished into blunt ends, adenylated and adaptors were ligated. The sample was then subject to a PEG/NaCl CA-purification to remove fragments lower than 200 base pairs (bp) and enriched by PCR before a final PEG/NaCl CA-purification. The automated protocol has replaced all MinElute™, Qiaquick PCR purification columns and the gel-cut with automated PEG/NaCl precipitation on CA-beads as previously described [6]. The ethanol precipitation in the standard mRNA sample preparation protocol has been replaced in the automated version with an EtOH/TEG precipitation on CA-beads. In all other aspects the manual and the automated sample preparations follows the mRNA sequencing sample preparation instruction (Cat# RS-930-1001) by the manufacturer (Illumina, San Diego, CA, USA). For more details regarding materials and reagents, see Text S1.

### Cell Cultivation

The glioblastoma cell line U-251MG (Prof. Bengt Westermark, Uppsala University) was cultivated at 37°C in a 5% CO_2_ environment in Minimum Essential Medium Eagle (EMEM) (Sigma-Aldrich) with an addition of 10% Fetal Bovine Serum (FBS;Invitrogen). The cells were harvested at 60–70% confluency.

### RNA Extraction

The cells were harvested and the RNA was immediately extracted using the RNeasy extraction kit according to the manufacturer instructions (Qiagen, Hilden, Germany). The isolated total RNA was analyzed using a 2100 Bioanalyzer (Agilent Technologies, Santa Clara, CA, USA) with the Bioanalyzer RNA 6000 Nano kit.

### Evaluation of RNA Precipitation using EtOH/TEG and CA-beads

The automated protocol takes 120 µl of EtOH/TEG precipitation solution to 25 µl of CA-beads resuspended in 38 µl of binding buffer (20 mM Tris-HCl pH 7,5, 1 M LiCl, 2 mM EDTA) and 2 µl of samples to capture the RNA. The beads with captured RNA were washed once with EtOH and the RNA was eluted using 10 µl of elution buffer (10 mM Tris-HCl).

The RNA precipitation efficiency was evaluated by varying the final concentration of EtOH and TEG while precipitating High and Low RiboRuler™ RNA ladders (Fermentas, Burlington, Canada): 200–6000 nucleotides, 100–1000 nucleotides respectively, and 20–100 nucleotides Small RNA marker (Abnova, Tapei city, Taiwan). The results were analyzed using a 2100 Bioanalyzer with the Bioanalyzer RNA 6000 Nano kit for the RiboRuler™ ladders and Small RNA kit for the Small RNA marker.

### Transcriptome Sample Preparation for Sequencing

A total amount of 3 µg per sample of high-quality total RNA (RNA integrity number = 10) was used as input material for the mRNA sample preparations. Samples, from the same biological material, were prepared in quadruplicates for both manual and automated preparations according to the mRNA sequencing sample preparation instruction (Cat# RS-930-1001) by the manufacturer (Illumina, San Diego, CA, USA). To assess the quality of the samples throughout the sample preparation each module of the protocols were monitored using a 2100 Bioanalyzer or Experion automated electrophoresis system (Bio-Rad Laboratories, Hercules, CA, USA). All sample preparation reagents were taken from the Illumina mRNA sample preparation kit or ordered from vendors specified in the mRNA sample preparation protocol, except for automation specific reagents: carboxylic acid beads used for precipitation; the EtOH/TEG and PEG/NaCl precipitation buffers. The final EtOH/TEG concentration used in the automated library preparation was 70% and 5% respectively. The final PEG/NaCl concentrations used were 15.6% PEG and 0.9 M NaCl except for the CA-beads purification after adapter ligation, which used a final PEG/NaCl concentration of 10.4% and 0.9 M respectively to remove fragments below 200 bp.

### Clustering and Sequencing

The clustering was performed on a cBot cluster generation system using an Illumina HiSeq single read cluster generation kit according to the manufacturer's instructions. One of the automated replicates (Aut3) failed in the clustering due to a malfunctioning pump on a cBot and was therefore sequenced individually in a separate sequencing run. The manual and automated library preparations were sequenced on an Illumina HiSeq 2000 as single-reads to 100 bp using 1 lane per sample on the same flow-cell (first sequencing run), except for Aut3 that failed in clustering and was run on a separate flow-cell using the same parameters (second sequencing run). All lanes were spiked with 1% phiX control library. The two sequencing runs were performed according to the manufacturer's instructions and generated a total of 477 million reads from the prepared libraries that passed the illumina Chastity filter; these reads were included in the study.

### Sequence Analysis

All sequences were analysed using the CASAVA v1.7 (Illumina, San Diego, CA, USA). The reads were aligned to the human genome reference Hg19 using Eland2 and variant detection was performed using the readBase method within the CASAVA software. Annotations from RefSeq, downloaded from UCSC Genome Browser, were used to assign features to genomic positions.

## Results

### RNA Purification

**Figure 1:**
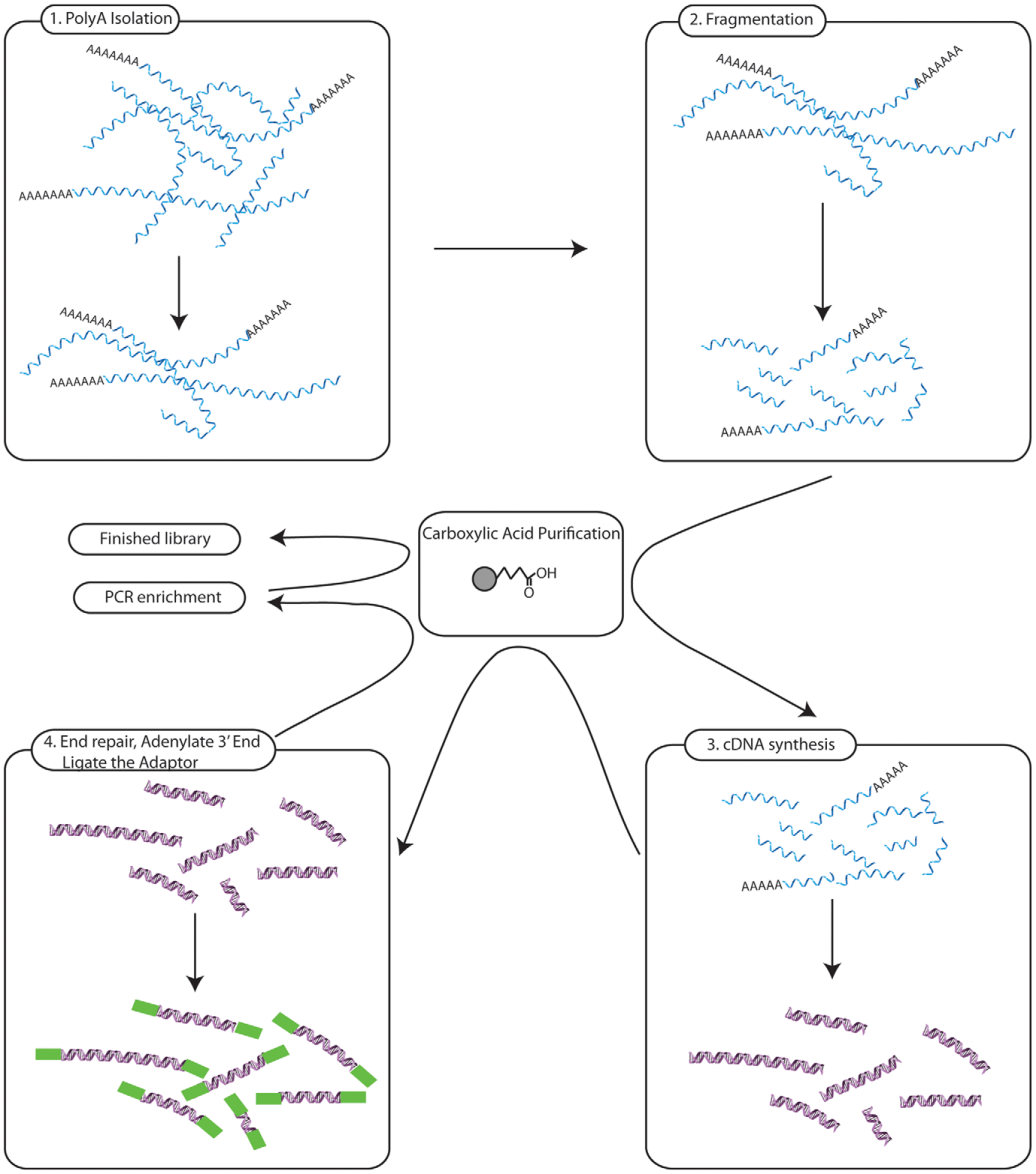
A schematic view of the automated process. Module 1: Isolation of poly-A containing mRNA from total RNA. Module 2: Fragmentation followed by precipitation on carboxylic acid coated beads (CA-purification). Module 3: cDNA synthesis of purified and fragmented mRNA. Module 4: End repair, 3′ adenylation, adaptor ligation and a CA-purification to remove fragments lower than 200 bp. The samples are then enriched by PCR before a final CA-purification.

**Figure 2:**
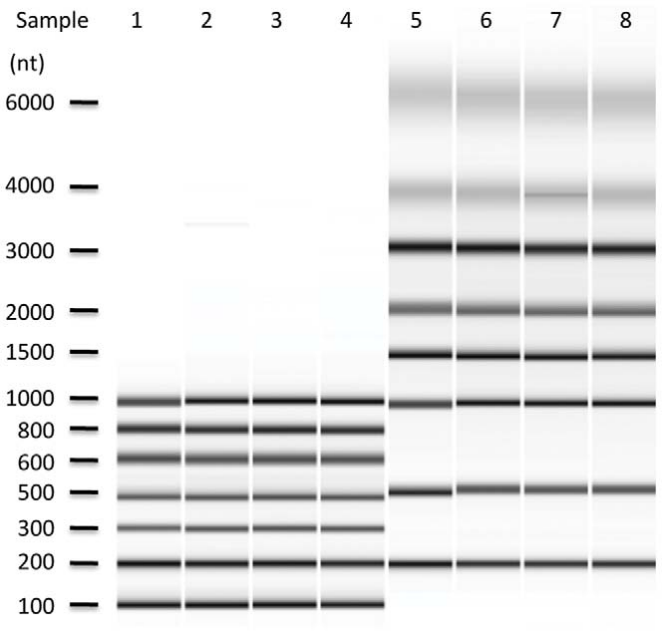
Precipitation of Low and High RiboRuler™. Bioanalyzer gel image showing precipitation of Low and High Riboruler™ ranging from 100 to 6000 nucleotides. Sample loaded from left to right are: Lane 1, Low RiboRuler™; Lane 2–4, triplicates of the precipitated Low RiboRuler; Lane 5, High RiboRuler™; Lane 6–8, triplicates of precipitated High RiboRuler™. The samples were analyzed using Bioanalyzer 6000 Nano kit.

The automation of the mRNA sample preparation protocol outlined in Figure 1 was established in modular fashion, to facilitate incorporation of future changes. The EtOH/TEG precipitation of RNA on CA-beads was evaluated using RNA ladders, spanning from 20 to 6000 nucleotides, to determine the robustness and RNA precipitation length cut-off. A titration of EtOH and TEG were performed (Figure S1) and by using the optimal final concentration of 70% EtOH and 5% TEG, a capture yield over 80% was obtained in the length interval of 100–6000 nucleotides (Table S1). The high reproducibility of the selected precipitation conditions is shown in Figure 2. Lowering either the EtOH or TEG concentration affects the yield of the precipitated RNA but not the length of the fragments that are precipitated. The minimum fragment length that could be precipitated was 40 nucleotides but the yield for fragments lower than 100 nucleotides were much reduced (data not shown).

### Sample Preparation

The automated and manual sample preparation follows the mRNA sequencing sample preparation instruction (Cat# RS-930-1001) by the manufacturer (Illumina) in every aspect unless previously specified.

**Figure 3:**
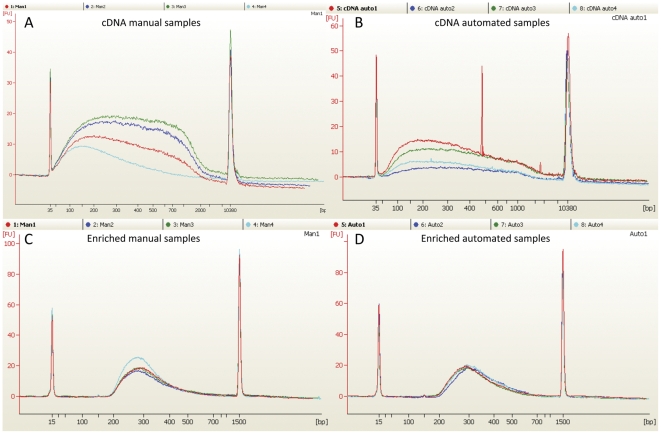
Length distribution and amount of manual and automated libraries after cDNA synthesis and enrichment by PCR. Samples 1–4 for each method are coloured in red, green, blue and cyan respectively. A: Manual samples after the cDNA synthesis. B: Automated samples after cDNA synthesis. The peak around 1000 bp for sample 1 (red curve) corresponds to a single bead remaining from the CA-purification. This peak has been removed from calculation of the total concentration. C: PCR enriched manual samples. D: PCR enriched automated samples. A and B is analyzed using the Bioanalyzer DNA High Sensitivity kit while C and D is analyzed using the Bioanalyzer DNA 1000 kit.

To be able to compare the automated and manual transcriptome preparations, quadruplicates of total RNA were prepared from the same biological material for each sample preparation. The library preparations were monitored using the 2100 Bioanalyzer after cDNA synthesis and PCR enrichments (Figure 3). Both the manual and automated preparation showed highly reproducible size distributions after cDNA syntheis (Figure 3A and 3B) and PCR amplification (Figure 3C and 3D) with a final yield of 13–17 ng/µl (Table S3). The yield after the cDNA synthesis is summarised in Table S2.

The throughput of the automated preparation was significantly greater than the manual preparation. Automated processing of 12 samples takes 10 hours and 40 minutes with approximately 1 hour and 15 minutes of hands-on time using pre-aliquoted reagents to prepare the robot. The manual preparation of 4 samples takes 13 hours, with approximately 6 hours of hands-on time. Enrichment and evaluation of the finished library preparations were equal in sample throughput for both automated and manual preparations and were therefore not included in this comparison.

### Analysis of Sample Preparation by Sequencing

The automated and manual sample preparations were sequenced on an Illumina HiSeq 2000 to be able to compare yield, quality, sensitivity and gene expression quantification. The manual sample preparation produced slightly more clusters than the automated sample preparation but both methods generated comparable percentage clusters passed filters, percentage of base calls above 30 (%Q>30) and number of reads, except for Aut3 which was sequenced on a separate flow-cell (Table 1). It is customary to spike in standard phiX library to 1% to be able to monitor the sequence run performance. The mean percentage phiX error rate for the first sequencing run was 2.06, which is above the allowed threshold of 2.0 specified by the manufacturer's, indicating a suboptimal sequencing run; a measure which is independent of the quality of loaded library preparations.

**Table 1:** Summary of information for the sequencing runs.

Sample	Preparation	Flow-cell	Lane	Conc (pmol)	Cluster density (K*/mm^2^)	PF Clusters (K*/mm^2^)	Clusters PF (%)	#Reads PF (M*)	%Q>30
U-251MG	Manual library 1	1	1	7	636	498	78.6	61.2	62.8
U-251MG	Manual library 2	1	2	7	592	472	79.8	58.0	62.5
U-251MG	Manual library 3	1	3	7	679	516	76.2	63.3	61.4
U-251MG	Manual library 4	1	4	7	709	528	74.8	64.9	60.8
U-251MG	Automated library 1	1	5	7	659	506	76.8	62.1	60.7
U-251MG	Automated library 2	1	6	7	655	501	76.6	61.5	61.0
U-251MG	Automated library 3	2	8	6	474	418	88.1	51.3	74.6
U-251MG	Automated library 4	1	8	7	566	450	79.6	55.3	61.0

*K = 10^3^, M = 10^6^.

**Figure 4:**
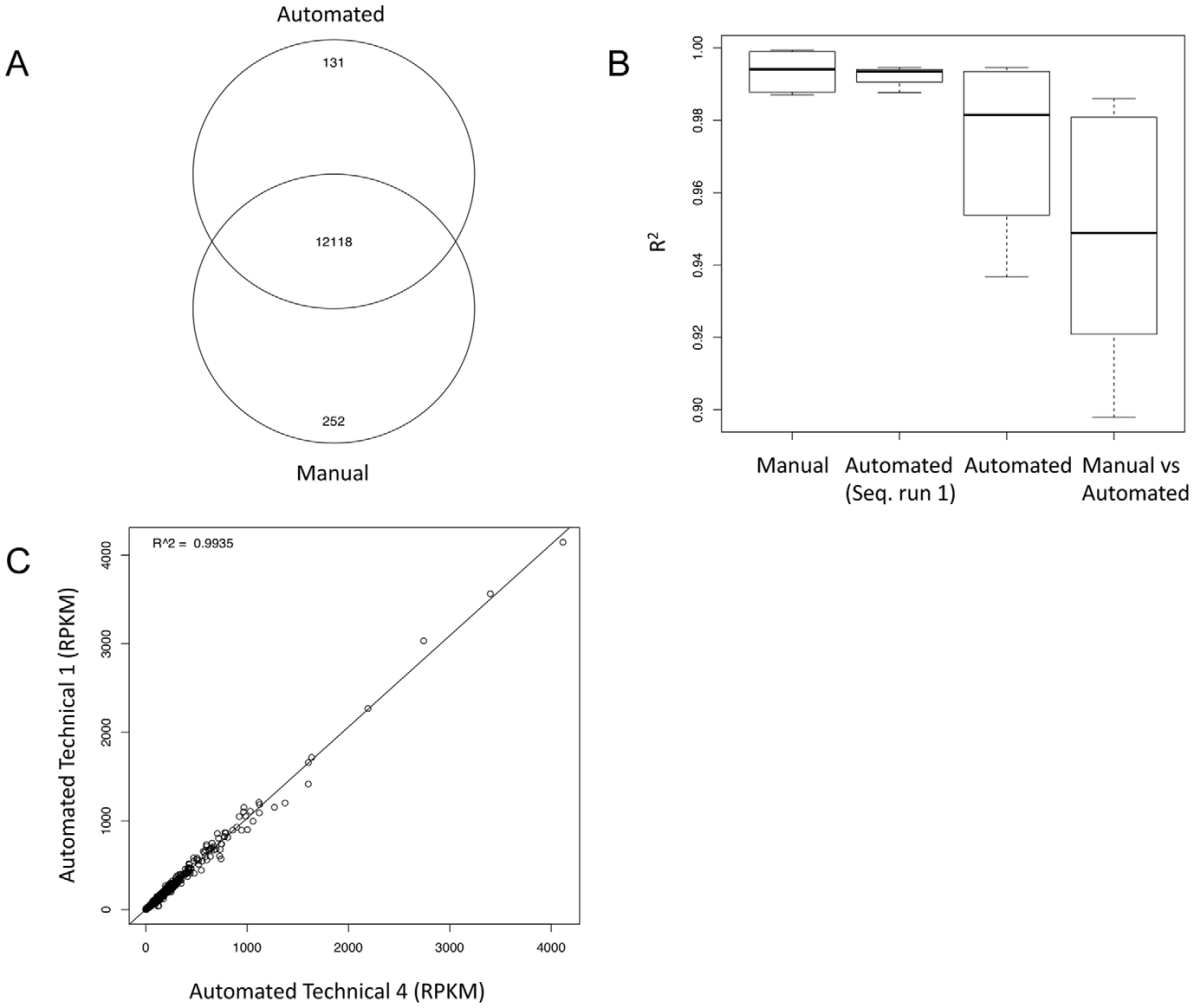
Comparison of gene expression levels and determination scores between the two methods. A: Venn diagram comparing number of expressed genes in both preparation methods. B: Boxplot of determination scores (R^2^) between the two methods and technical replicates. C: Comparison of two automated technical replicate RNA-Seq determinations, measured in RPKM values.

We define an expressed gene as having a normalised exonic read density value above 0.3, which is measured in reads per kilobase of exon per million mapped sequence reads (RPKM; [12]). The total number of expressed genes found within all replicates for each preparation was similar in both the automated and manual sample preparations. Of all expressed genes present in both the automated and the manual sample preparations 96.9% can be found in all replicates in both libraries (Figure 4A). The majority of uniquely expressed genes in the automated and manual preparation were weakly expressed with a median RPKM value of 0.65 and 0.45 respectively (Figure S3). The distribution of expressed genes within each preparation and replicate were similar (Figure S2).

The correlation of RPKM values between replicates within the automated and manual preparations were excellent with the mean coefficient of determination (R^2^) value of 0.974 and 0.994 respectively (Figure 4B). If the Aut3 sample, which was sequenced in the second sequencing run is excluded from the analysis the mean R^2^ value for the automated samples preparation was 0.992. There were good correlations between manual and automated sample preparation replicates with mean R^2^ value of 0.946 (Figure 4C).

## Discussion

This study describes an automated transcriptome preparation for massively parallel sequencing. The automated library preparation procedure was evaluated by comparing it to standard manual library preparation from the same biological material. The evaluation shows that the automated preparation has significantly higher throughput compared to the manual preparation. The two library preparations were comparable in percentage clusters passed filters, %Q>30, and number of reads when sequenced on the same flow-cell. Having to sequence one of the technical replicates in a separate sequencing run, due to a malfunction on the cluster station, gave lower correlation for this replicate compared to the technical replicates sequenced on the same flow-cell. Although, this does not prove that there is greater variation between sequencing runs than within samples sequenced on the same flow-cell, it does suggest that care should be taken to sequence as much as possible within the same sequencing run [4], [13].

Due to the fast evolution of massively parallel sequencing technologies the sequence capacity is likely to increase in the future, further emphasizing the need for robust, scalable sample preparations. Therefore, the automated protocol is organized into separate modules to be able to accommodate updates in library preparations and using different sources of input material i.e. total RNA, ribosome depleted RNA, mRNA or cDNA (Figure 1). This will make sure that the automated protocol easily can be scaled up and adapted to new upgrades in library preparation such as the new TruSeq RNA™ sample preparation protocol. Currently, we are working on further increasing the capacity of the automated RNA protocol to being able to handle 24 samples at a time with only a 10% increase in total library preparation time.

Precipitating RNA using standard ethanol precipitation or spin columns are tedious, difficult to automate and scale up. Our novel EtOH/TEG precipitation procedure using CA-beads as a solid support is readily automated, fast and reproducible. We have shown that we can precipitate RNA fragments sized between 100–6000 nucleotides with yields above >80% making it an attractive general method for isolating RNA from a solution

In conclusion, this is the first demonstration of an automated transcriptome preparation for massively parallel sequencing performed for the Illumina HiSeq 2000 instrument. The protocol is robust, user friendly and has 14 times higher sample throughput than the manual sample preparation. It is flexible and can easily be updated to accommodate updates to the mRNA library preparation protocol and can also be extended to other massively parallel platforms. The EtOH/TEG precipitation is readily automated for rapid and easy handling and can be used in general, whenever RNA needs to be isolated from a solution. The automated transcriptome preparation protocol here described will alleviate the bottleneck of sample preparation in RNA sequencing.

## Supporting Information

Figure S1 **Titration of EtOH and TEG on High RiboRuler™.** Bioanalyzer gel image showing titration and precipitation effect on High RiboRuler™. Precipitation solution used for samples from left to right are: Lane 1–20% EtOH and 1% TEG; Lane 2–20% EtOH and 5% TEG; Lane 3–20% EtOH and 15% TEG; Lane 4–50% EtOH and 1% TEG; Lane 5–50% EtOH and 5% TEG; Lane 6–50% EtOH and 15% TEG; Lane 7–70% EtOH and 1% TEG, Lane 8–70% EtOH and 5% TEG; Lane 9 - High RiboRuler™. The samples were analyzed using Bioanalyzer 6000 Nano kit.
(TIF)

Figure S2 **Venn diagram comparing number of expressed genes for each preparation method.** A–D technical replicates within each preparation method.
(TIF)

Figure S3 **RPKM values of uniquely expressed genes from the automated and manual preparations.**
(TIF)

Table S1 **Yield for precipitation of Low and High RiboRuler™ in triplicates.**
(TIF)

Table S2 **cDNA concentration between 200 and 1000 bp for manual and automated sample preparation, respectively.** The samples were analysed using Bioanalyzer DNA High Sensitivity kit.
(TIF)

Table S3 **Final library DNA concentration between 220 and 700 bp for manual and automated sample preparation, respectively.** The samples were analysed using Bioanalyzer DNA 1000 kit.
(TIF)

Text S1 **Detailed description of automated protocol and material and reagents.**
(DOCX)
