# Supplementary figures and images for: Scalable Transcriptome Preparation for Massive Parallel Sequencing
Source: PLoS One. 2011 Jul 7;6(7):e21910. doi: 10.1371/journal.pone.0021910 (PMC3131396; doi:10.1371/journal.pone.0021910)

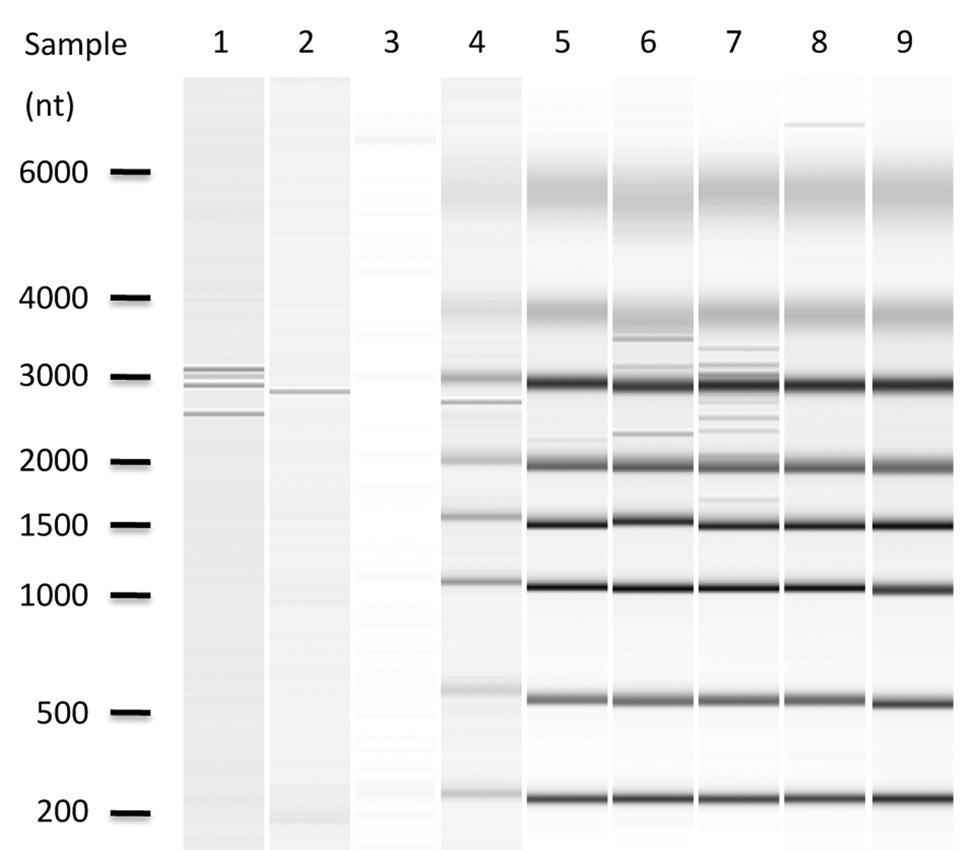

Supplement: Figure S1 — Titration of EtOH and TEG on High RiboRuler™. Bioanalyzer gel image showing titration and precipitation effect on High RiboRuler™. Precipitation solution used for samples from left to right are: Lane 1–20% EtOH and 1% TEG; Lane 2–20% EtOH and 5% TEG; Lane 3–20% EtOH and 15% TEG; Lane 4–50% EtOH and 1% TEG; Lane 5–50% EtOH and 5% TEG; Lane 6–50% EtOH and 15% TEG; Lane 7–70% EtOH and 1% TEG, Lane 8–70% EtOH and 5% TEG; Lane 9 - High RiboRuler™. The samples were analyzed using Bioanalyzer 6000 Nano kit. (TIF) [file pone.0021910.s001.tif]

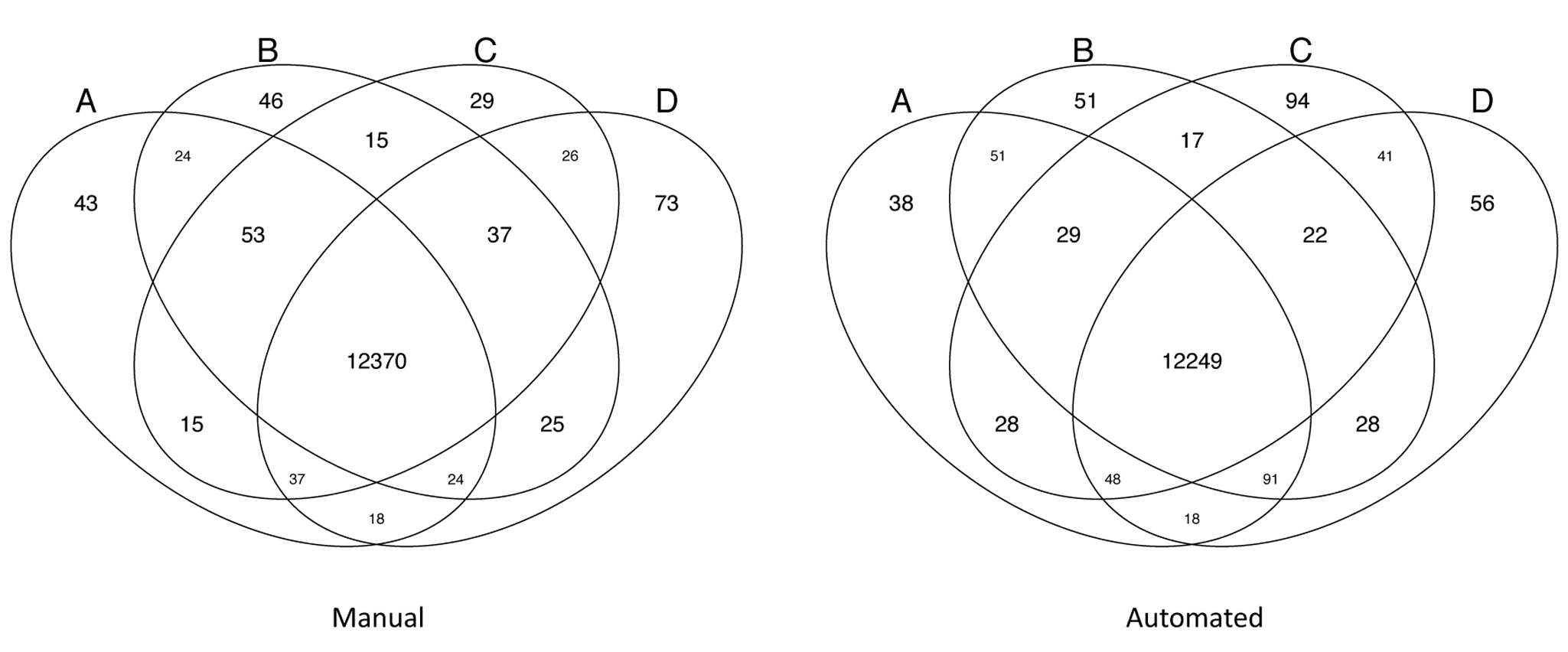

Supplement: Figure S2 — Venn diagram comparing number of expressed genes for each preparation method. A–D technical replicates within each preparation method. (TIF) [file pone.0021910.s002.tif]

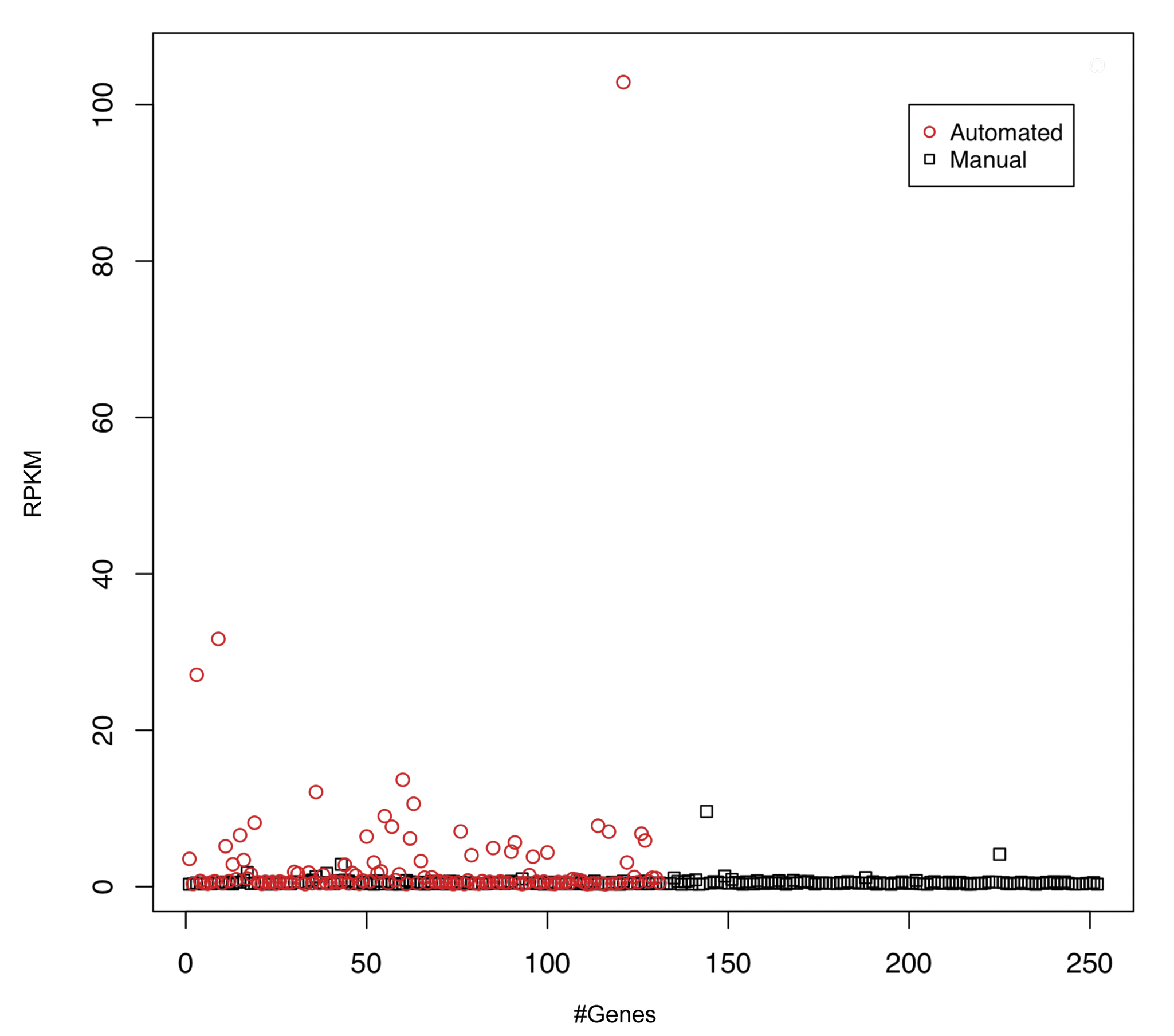

Supplement: Figure S3 — RPKM values of uniquely expressed genes from the automated and manual preparations. (TIF) [file pone.0021910.s003.tif]

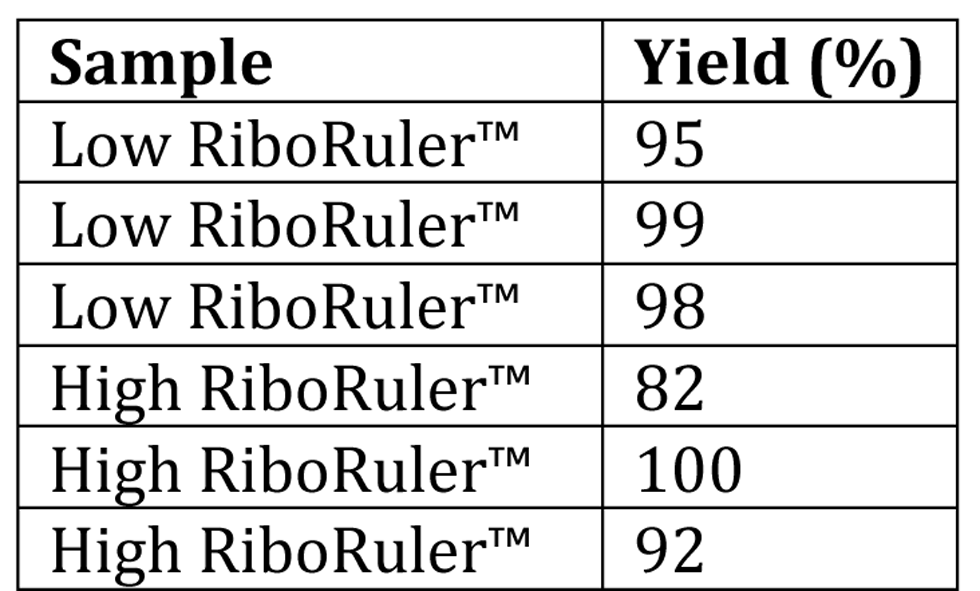

Supplement: Table S1 — Yield for precipitation of Low and High RiboRuler™ in triplicates. (TIF) [file pone.0021910.s004.tif]

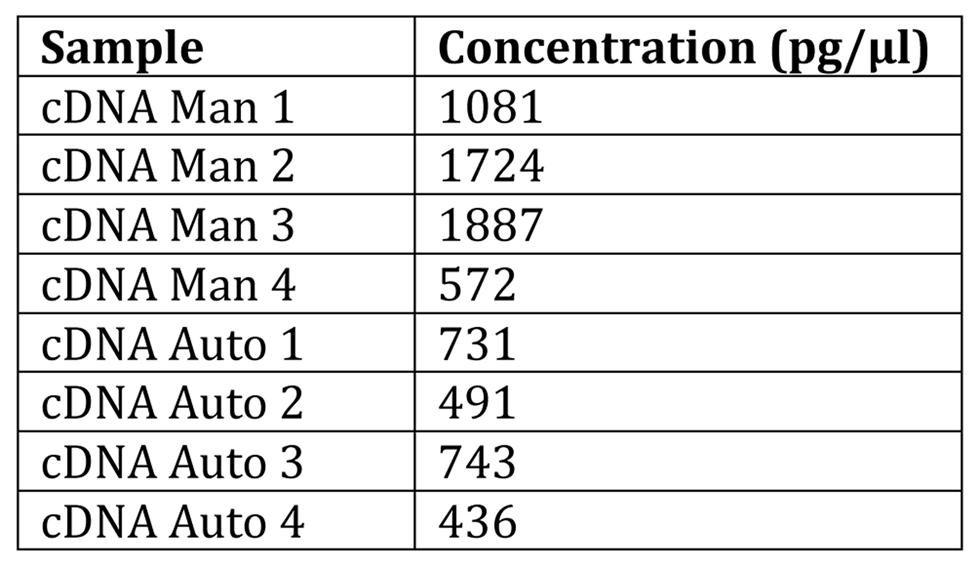

Supplement: Table S2 — cDNA concentration between 200 and 1000 bp for manual and automated sample preparation, respectively. The samples were analysed using Bioanalyzer DNA High Sensitivity kit. (TIF) [file pone.0021910.s005.tif]

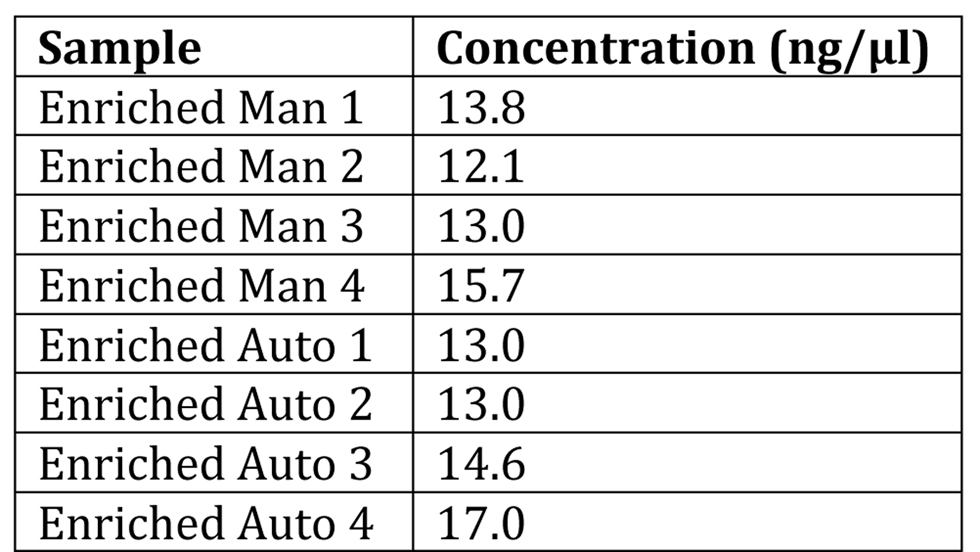

Supplement: Table S3 — Final library DNA concentration between 220 and 700 bp for manual and automated sample preparation, respectively. The samples were analysed using Bioanalyzer DNA 1000 kit. (TIF) [file pone.0021910.s006.tif]
