# Supplementary material for: Scalable Transcriptome Preparation for Massive Parallel Sequencing
Source: PLoS One. 2011 Jul 7;6(7):e21910. doi: 10.1371/journal.pone.0021910 (PMC3131396; doi:10.1371/journal.pone.0021910)
Supplement: Text S1 — Detailed description of automated protocol and material and reagents. (DOCX) [file pone.0021910.s007.docx]

# Text S1

## Material and reagents

**1.** **PolyA isolation:** Sera-Mag Magnetic Oligo(dT) beads (Illumina, San Diego, CA USA), Bead Binding Buffer (Illumina), Bead Washing Buffer (Illumina), 10mM Tris-HCl.

**2.** **Fragmentation:** 5X Fragmentation Buffer (Illumina), Fragmentation Stop Solution (Illumina), Binding Buffer (20mM Tris-HCl, 1M LiCl, 2mM EDTA), Precipitation Buffer (93% EtOH, 6.7% TEG), 80% Ethanol, 10mM Tris-HCl, RNase-free water, Dynabeads® MyOne™ carboxylic acid paramagnetic beads (Invitrogen, Carlsbad, CA USA).

**3.** **cDNA synthesis**: 25mM dNTP Mix (Illumina), Random Primers (Illumina), RNase Inhibitor (Illumina), SuperScript II (Invitrogen), 100mM DTT (Invitrogen), 5X First Strand Buffer (Invitrogen), GEX Second Strand Buffer (Illumina), RNaseH (Illumina), DNA Pol I (Illumina), Buffer EB (Qiagen, Valencia, CA USA), Precipitation Buffer (1,5M NaCl, 25% TEG), 80% Ethanol, Dynabeads® MyOne™ carboxylic acid paramagnetic beads (Invitrogen).

**4.** **End repair, adenylation of 3' ends, adaptor ligation**: 10X End Repair Buffer (Illumina), 25mM dNTP Mix (Illumina), T4 DNA Polymerase (Illumina), Klenow DNA Polymerase (Illumina), T4 PNK, A-Tailing Buffer (Illumina), 1mM dATP, Klenow exo (3' to 5' exo minus) (Illumina), 2X Rapid T4 DNA Ligase Buffer (Illumina), PE Adaptor Oligo Mix (Illumina), T4 DNA Ligase (Illumina).

**5**. **Gel-cut**: Buffer EB (Qiagen), Precipitation Buffer (1,5M NaCl, 12,5% TEG), 80% Ethanol, Dynabeads® MyOne™ carboxylic acid paramagnetic beads (Invitrogen).

**6.** **PCR**: 5X Phusion Buffer and Phusion DNA Polymerase (Finnzymes Oy, Finland), PCR Primer InPE 1.0 and 2.0 and specific index primers (Illumina), 25mM dNTP Mix (Illumina), Ultra Pure Water.

**7.** **PCR product purification**: Buffer EB (Qiagen), Precipitation Buffer (1,5M NaCl, 25% TEG), 80% Ethanol, Dynabeads® MyOne™ carboxylic acid paramagnetic beads (Invitrogen).

## Automated protocol

The protocol used for automation is briefly described below. For a more detailed protocol see original protocol from the manufacturer Illumina (Cat# RS-930-1001).

### PolyA isolation

A total amount of 3 µg per sample was used as input material for the automated sample preparation. The sample preparation protocol begun with a poly-A isolation of mRNA with Sera-Mag Magnetic Oligo(dT) beads (Illumina). The RNA sample was heated for 5 min at 65°C to disrupt the secondary structures before the RNA sample was incubated 5 min with the Sera-Mag Magnetic Oligo(dT) beads. The beads were subject to repeated washes and eluted for 2 min at 80°C in 50µl of 10 mM Tris-HCl. 50µl of Binding Buffer (Illumina) was added to the elute and the sample was heated for 5 min at 65°C. The sample was then incubated with the beads one more time, washed and eluted in 17µl of 10 mM Tris-HCl at 80°C for 2 min.

### Fragmentation

The polyA isolated sample was fragmented using divalent cations under elevated temperature. The mRNA was mixed with 4 µl 5X Fragmentation Buffer (Illumina) and incubated at 94°C for 5 min before 2 µl of Fragmentation Stop Solution (Illumina) was added. The automated sample was then subject to a purification using CA-beads and precipitation buffer (93% EtOH, 6.7% TEG), washed using 80% ethanol and eluted in 11.1µl RNase-free water while the manual prepared samples were purified according to manufactures protocol.

### First and second strand cDNA synthesis

Random primers were added to the purified fragmented sample and incubated at 65°C for 5 minutes. Thereafter was SuperScript II (Invitrogen) reaction mix added together with dNTP mix (Illumina) and RNase inhibitor (Illumina) and incubated with the following program: 25°C for 10 minutes, 42°C for 50 minutes, 70°C for 15 minutes and hold at 4°C. The second strand synthesis starts with the addition of ultra pure water to the first strand cDNA synthesis mix and then the addition of GEX Second Strand Buffer (Illumina) and dNTP mix (Illumina). The sample was incubated for 5 min at 4°C before RNaseH (Illumina) and DNA PolI (Illumina) was added and incubated at 16°C for 2.5 hours. The automated samples were then purified using CA-beads and precipitation buffer (1,5M NaCl, 25% TEG), washed with 80% ethanol and eluted in EB buffer as described by *Lundin et al[*[*1*](#_ENREF_1)*]*., while the manual prepared samples were purified according to manufactures protocol.

### End repair, adenylation of 3' ends, adaptor ligation

The overhang of the cDNA sample ends were polished into blunt ends by addition of water, 10X End Repair Buffer (Illumina), dNTP mix (Illumina), T4 DNA Polymerase (Illumina), Klenow DNA Polymerase (Illumina) and T4 PNK (Illumina) to the eluted DNA and incubated at 20°C for 30 minutes. The 3’ ends were adenylated by the use of Klenow exo (3’ to 5’ exo minus) (Illumina), dATP (Illumina) and A-Tailing Buffer (Illumina) and incubated at 37°C for 30 minutes. The adaptor ligation starts with addition of 2X Rapid T4 DNA Ligase buffer (Illumina), T4 DNA Ligase (Illumina) and Adaptor Ologo mix (Illumina) to the DNA sample and incubated for 15 minutes at room temperature. After each of the three processes the samples were purified using CA-beads and precipitation buffer (1,5M NaCl, 25% TEG), washed with 80% ethanol and eluted in EB buffer as described by *Lundin et a*l*[*[*1*](#_ENREF_1)*]*.

### Gel-cut

The purification of fragments around 200 bp on agarose gel was replaced with an automated purification using CA-beads, 80% ethanol and precipitation buffer (1,5M NaCl, 12,5% TEG) as described by *Lundin et al[*[*1*](#_ENREF_1)*].*

### PCR and purification

The purified cDNA samples were enriched by PCR according to preparation instructions (Cat# RS-930-1001) from the manufacturer (Illumina). The enriched sample was subject to a final purification using CA-beads and precipitation buffer (1,5M NaCl, 25% TEG), washed with 80% ethanol and eluted in EB buffer as described by *Lundin et al[*[*1*](#_ENREF_1)*].*

**Supplemental References**

1. Lundin S, Stranneheim H, Pettersson E, Klevebring D, Lundeberg J (2010) Increased throughput by parallelization of library preparation for massive sequencing. PLoS One 5: e10029.
